# Posttranslational and Therapeutic Control of Gasdermin-Mediated Pyroptosis and Inflammation

**DOI:** 10.3389/fimmu.2021.661162

**Published:** 2021-04-02

**Authors:** Fabian A. Fischer, Kaiwen W. Chen, Jelena S. Bezbradica

**Affiliations:** ^1^ Kennedy Institute of Rheumatology, NDORMS, University of Oxford, Oxford, United Kingdom; ^2^ Immunology Programme and Department of Microbiology & Immunology, Yong Loo Lin School of Medicine, National University of Singapore, Singapore, Singapore

**Keywords:** pyroptosis, gasdermins, cell death, post-translational modifications, therapeutics, phosphorylation, inflammation

## Abstract

Pyroptosis is a proinflammatory form of cell death, mediated by membrane pore-forming proteins called gasdermins. Gasdermin pores allow the release of the pro-inflammatory cytokines IL-1β and IL-18 and cause cell swelling and cell lysis leading to release of other intracellular proteins that act as alarmins to perpetuate inflammation. The best characterized, gasdermin D, forms pores *via* its N-terminal domain, generated after the cleavage of full length gasdermin D by caspase-1 or -11 (caspase-4/5 in humans) typically upon sensing of intracellular pathogens. Thus, gasdermins were originally thought to largely contribute to pathogen-induced inflammation. We now know that gasdermin family members can also be cleaved by other proteases, such as caspase-3, caspase-8 and granzymes, and that they contribute to sterile inflammation as well as inflammation in autoinflammatory diseases or during cancer immunotherapy. Here we briefly review how and when gasdermin pores are formed, and then focus on emerging endogenous mechanisms and therapeutic approaches that could be used to control pore formation, pyroptosis and downstream inflammation.

## Introduction: Gasdermin-Mediated Cell Death as a Driver of Inflammation

Apoptosis is traditionally viewed as a non-inflammatory form of caspase-dependent programmed cell death (1). During apoptosis, caspase-mediated inactivation of innate immune signaling molecules and the preservation of membrane integrity ensures that apoptotic cells remain immunologically silent (albeit in some cases, cells can transition from apoptosis to other, more inflammatory forms of cell death) (2). Pyroptosis and necroptosis are inflammatory types of programmed cell death, driven by dedicated membrane pore-forming proteins, gasdermins (pyroptosis) or MLKL (necroptosis), respectively. During pyroptosis and necroptosis, the cell loses plasma membrane integrity, ruptures and uncontrollably releases the cytosolic content including cytosolic alarmins that drive inflammation (3).

Pyroptosis can drive both microbe-induced and sterile inflammation and can be beneficial or pathological. For example, during infection, gasdermin D (GSDMD) is cleaved by caspase-1, caspase-8 and caspase-11 (caspase 4/5 in humans), to release its membrane pore-forming fragment and induce pyroptotic death of infected cells (4–10). This is beneficial, as pyroptosis releases alarmins and destroys the cellular niche for pathogen replication (11, 12). But if excessive, pyroptosis can cause immunopathology, and in fact, caspase-1/11, caspase-11 and GSDMD-deficient mice are protected from mouse models of lethal LPS- and TNF-induced shock and polymicrobial sepsis (4, 13–20). Gasdermins can also drive sterile inflammation, which can also be beneficial or pathological. For example, gasdermin B (GSMDB) and gasdermin E (GSDME) are cleaved into their membrane pore-forming fragments by the enzymes granzyme A and B, respectively, when granzymes are delivered directly into the tumor cell during the attack by cytotoxic T cells. Once cleaved, GSDMB and GSMDE induce the death of tumor cells by pyroptosis, resulting in low-grade local inflammation that is essential for successful clearance of tumors by myeloid cells (21, 22). If however GSDME-mediated pyroptosis of tumor cells is excessive, as seen in response to chimeric antigen receptor (CAR) T cell therapy, dying cells release alarmins, activate caspase-1/GSDMD pathway in recruited macrophages, and cause systemic cytokine release syndrome, a common complication of CAR T cell therapy (23). Similarly, several chemotherapy drugs that were designed to induce non-inflammatory apoptosis of tumors, end up causing systemic pathology, by activating the apoptotic caspase-3, which can cleave GSDME into its pore-forming fragment. These systemic drugs thus induce pyroptosis not only in GSDME-positive tumors, but also in other GSDME-expressing healthy cells, leading to wide-spread inflammation, tissue damage and weight loss (24). Select chemotherapeutic agents have also been described to activate caspase-8-dependent GSDMC activation in tumor cells (25), or GSDMD activation in myeloid cells (10), however, whether myeloid GSDMD activation promotes or dampens tumor growth *in vivo* remains unclear. Finally, GSDMD deletion is protective in several mouse models of inherited and acquired sterile inflammatory diseases, such as Familial Mediterranean Fever, Neonatal-Onset Multisystem Inflammatory Disease, Experimental Autoimmune Encephalomyelitis, or liver damage (26–31). Therefore, understanding how and where gasdermins are activated and how pyroptosis can be regulated, will provide new opportunities for the control of inflammation.

Many excellent reviews have discussed in detail events leading to gasdermin activation (32, 33). Here we will focus briefly on how and when gasdermin pores are formed, and then focus on emerging endogenous posttranslational mechanisms and therapeutic approaches that could be used to control gasdermin pore formation, pyroptosis and downstream inflammation.

## Gasdermins: Expression, Function and Localization

Gasdermins are a family of newly described proteins that are emerging as key players in inflammation. Humans express six gasdermin family proteins: *GSDMA*, *GSDMB*, *GSDMC*, *GSDMD*, *GSDME* (formerly called *DFNA5*) *and PJVK*. In contrast, mice and rats do not express *Gsdmb*, but instead, have three GSDMA homologs (*Gsdma1–Gsdma3*) and four GSDMC homologs (*Gsdmc1–Gsdmc4*) (34). Gasdermin family proteins are differentially expressed in various tissues and we are only beginning to understand the biological functions of these proteins (33). Of which, the pore-forming properties of GSDMD and GSDME in myeloid cells and tumors have gained considerable attention recently and will be the focus of this review.

**Figure 1:**
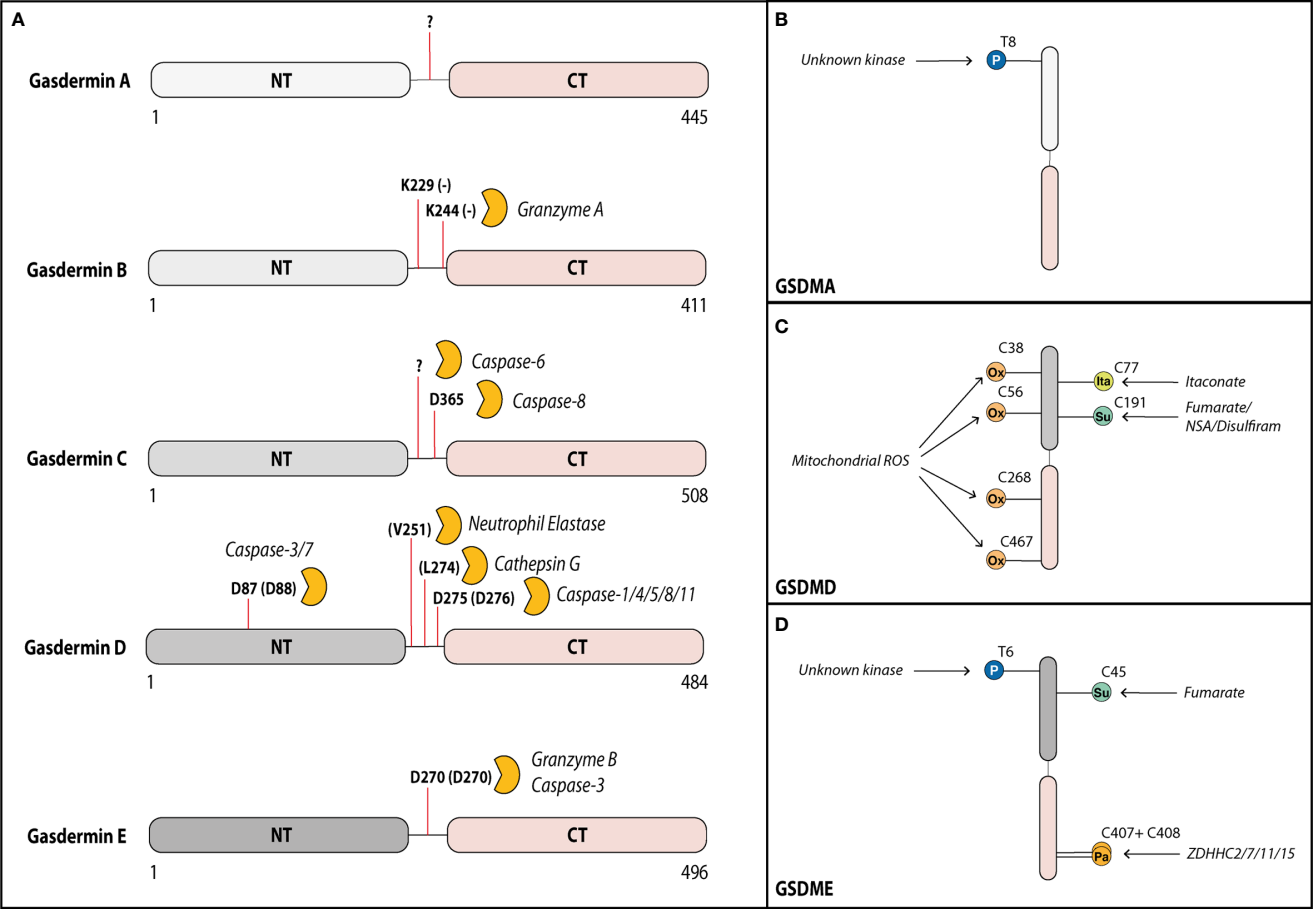
Gasdermins can be cleaved by various proteases in their linker region. Activity of gasdermins is regulated by cleavage and by post-translational modifications. **(A)** Gasdermin A can form membrane pores after cleavage of the linker domain, but the cleaving protease remains unknown. In tumor cells, Gasdermin B can be cleaved by granzyme A from cytotoxic T cells at Lys229 and Lys244 into the pore forming fragment. In several cell types including most myeloid cells, gasdermin D can be cleaved by multiple proteases at Asp275 (mouse Asp276) leading to its activation, but it can additionally be cleaved at Asp87 (mouse Asp88) by caspase-3 and -7 inactivating it during apoptosis. In neutrophils, gasdermin D can also be cleaved by neutrophil elastase and cathepsin D. In response to some chemotherapy drugs, gasdermin C can be cleaved by caspase-6 (at unknown site) and caspase-8 (at Asp365) into the pore forming fragment. Gasdermin E can be cleaved by granzyme B or caspase-3 at Asp270 leading to activation. Activity of gasdermins is also regulated by several post-translational modifications. **(B)** Gasdermin A can be phosphorylated (P) by an unknown kinase at Thr8, supporting its pore-forming capacity. **(C)** Gasdermin D is oxidized (Ox) at multiple residues (Cys38, Cys56, Cys268 and Cys467) by reactive oxygen species from the mitochondria promoting its activation. Prolonged LPS exposure of macrophages results in binding of itaconate at Cys77 preventing gasdermin D cleavage. Gasdermin D can also be succinated (Su) at Cys191 by the metabolic product fumarate or by covalent binding of the cysteine-reactive drugs necrosulfonamide (NSA) or disulfiram, which prevents its oligomerization. **(D)** Similar to gasdermin A, gasdermin E is phosphorylated at Thr6 promoting its pore formation. Gasdermin E is inhibited by succination at Cys45. During the activation, the palmitoyltransferases ZDHHC2, -7, -11 and -15 palmitoylate (Pa) gasdermin E at Cys407 and Cys408 promoting the dissociation of the GSDME-NT from GSDME-CT.

Gasdermins contain a cytotoxic N-terminal domain (GSDM-NT) and an autoinhibitory C-terminal domain (GSDM-CT), connected by a linker region that harbors a protease cleavage site (33). Microbial infection or cellular stress promotes the assembly of a cytosolic multiprotein inflammasome complex, which serves as a platform to activate inflammatory caspases, caspase-1 and -11 (caspase-4/5 in humans). These activated caspases as well as the proteases neutrophil elastase and cathepsin G cleave GSDMD within its linker region and liberate the cytotoxic GSDMD-NT to trigger plasma membrane damage and cell lysis by pyroptosis (**Figure 1A**) (4–6, 35, 36). Interestingly, the apoptotic executioner caspases-3 and-7 can cleave gasdermin D outside of the linker region at Asp87 leading to its inactivation and thereby negative regulation of pyroptosis (37). More recently, blockade of NF-κB and MAPK signaling, or perturbation in RIPK1 post-translational modifications by bacterial effectors or chemotherapeutic drugs were also demonstrated to promote GSDMD cleavage (8–10). Surprisingly, GSDMD cleavage under such circumstances occurred largely independently of inflammatory caspase-1/11, but instead, is mediated *via* apoptotic caspase-8. While new and exciting functions of GSDMD are mainly characterized in myeloid cells, the role of GSDME in host defense remains poorly characterized and is mainly characterized in tumor cells, where cleavage of GSDME by apoptotic caspase-3 and -7 or granzyme B switches tumor cell apoptosis to pyroptosis (7, 22, 24).

## Gasdermin Pore Formation, Repair and Cell Lysis Are Distinct and Regulated Events

A caspase-1-dependent cell death was initially described during *Salmonella* Typhimurium infection. Infected cells displayed cell death hallmarks such as release of the intracellular enzyme LDH, uptake of cell-impermeable dyes and exposure of phosphatidylserine from the inner to the outer leaflet of the plasma membrane (3, 38). The discovery of GSDMD as the cell death executioner explained all these features, owing to its ability to form pores in the plasma membrane leading to activation of several intracellular processes and resulting in cell swelling and death (4–6). However, the exact steps leading to pore formation and death, and the mechanisms in place to control these steps, remained poorly understood. Recent literature suggests that pyroptosis is a highly chronological and fine-tuned process that can be separated into sequential, highly regulated events. The main events in the process are GSDMD pore formation, ion fluxes, cellular death, and cell rupture. The following section dissects pyroptosis into these steps.

### Gasdermin Pore Formation

After cleavage of GSDMD at Asp275 (human) or Asp276 (mouse) by caspases, the N-terminal fragment (GSDMD-NT) localizes to the plasma membrane where it can bind to phospholipids, such as phosphatidylinositol phosphates or phosphatidyl serine on the inner leaflet of the plasma membrane (39, 40). Once at the membrane, the N-terminal fragments oligomerize, in a process dependent on a cysteine residue at position 192 in humans (Cys191 in mice), to form a functional pore (39). An elegant genetic screen by Evavold et al. (41), designed to identify regulators of pyroptosis downstream of GSDMD cleavage, showed that the GSDMD oligomerization is not a passive event but is regulated downstream of the Ragulator-Rag complex, typically known for its metabolic control of mTORC1 pathway. The components of the Ragulator-Rag complex, such as RagA or RagC, were dispensable for GSDMD trafficking to the cell membrane, but were essential for GSDMD-NT oligomerization and pore formation. During pyroptosis, GSDMD pore formation is typically followed by the loss of mitochondrial function (42), cell ballooning (42) and finally cell rupture (all discussed below). GSDMD-NT translocated to the plasma membrane in RagA-deficient cells but maintained membrane permeability (measured by propidium iodide (PI) uptake), mitochondrial function, and cell morphology, suggesting a role of the Ragulator-Rag complex in GSDMD pore formation itself and not in other, downstream events leading to cell death (41). It remains to be investigated whether any component of the Ragulator-Rag-mTORC1 pathway directly binds to GSDMD-NT to support oligomerization or if an intermediate interaction partner exists that exerts this action. LPS priming has been shown so activate mTORC1; hence, we speculate that the inflammasome priming step already puts the cell in a state in which it is prepared to commit cell death if needed (43).

### Ion Fluxes and Membrane Repair

Assembled GSDMD pores can measure up to 20 nm in diameter depending on the study and on the cellular system used (39, 44–47). These pores allow secretion of smaller intracellular proteins such as IL-1β (17 kDa) but do not permit the secretion of larger proteins such as a LDH (140 kDa) or the inflammation mediator HMGB1 (tetramer of 150 kDa), which were previously thought to be released *via* the gasdermin pores (44, 48, 49). These bigger mediators are instead released after cell lysis and the cellular content released (50). GSDMD pores not only function as protein secretion channels but also allow trafficking of nucleotides and act as non-selective ion channels. Shortly after pore assembly, extracellular Ca^2+^-ions enter the cell through the pore (42, 48, 51, 52). This ion influx triggers several processes in the cell. First, it activates the Endosomal Sorting Complexes Required for Transport (ESCRT) proteins I and III, which assemble at the plasma membrane to remove gasdermin pores by encapsulating them into vesicles. When successful, membrane integrity is restored, and cell lysis and IL-1β secretion are prevented (51). Intriguingly, recent analyses of ESCRT-produced vesicles during necroptosis revealed that they contain the pore-forming MLKL, active caspases and both full length and cleaved IL-1β, among other proteins (53, 54). This suggests that a similar repair mechanism and subsequent vesicle release might exist during Gasdermin-mediated pore formation in order to release these pro-inflammatory mediators, but this remains to be tested.

Contrary to the membrane repair and rescue role, Ca^2+^-ion influx through GSDMD pores can also have a pathologic consequence for the cell and the organism. For example, Ca^2+^-ion influx through GSDMD pores activates the Ca^2+^-dependent transmembrane protein 16F (TMEM16F), a membrane phospholipid scramblase, which enhances the presence of phosphatidylserine (PS) in the outer leaflet of the plasma membrane (52, 55). Once exposed, PS activates the initiator of coagulation called tissue factor, leading to life-threatening disseminated intravascular coagulation often seen in bacterial endotoxemia (52, 56). TMEM16F activation also causes a change in the cellular ion currents, at least in part due to the efflux of Cl^-^ ions (55), further contributing to the loss of ion homeostasis and cell death. Ca^2+^-ion influx during bacterial endotoxemia also activates STING (TMEM173) on the ER membrane. Activated STING then binds to and activates the calcium channel ITPR1 to trigger further Ca^2+^-release from ER stores. Elevated Ca^2+^ contributes to the activation of inflammatory caspases-1/11 or -8 (depending on the pathogen), leading to further GSMDM cleavage, activation of tissue factor, and lethal coagulation in bacterial sepsis (57). Finally, elevated Ca^2+^-levels trigger lipid peroxidation of cytoplasmic membrane lipids, by the enzyme PLCγ1, contributing to progression to pyroptosis and inflammation in polymicrobial sepsis (20, 52). While Ca^2+^-ions are taken up, nucleotides like ATP are released through the pore (48). Next to having an impact on the cellular energetic status, it was suggested that ATP release activates the ion channel P2X7 leading to increased uptake of Ca^2+^ and further progression to pyroptosis (58). Interestingly, both Ca^2+^- and ATP-mediated pyroptosis appeared to be blocked by extracellular Mg^2+^-ions, which are known to chelate ATP and prevent increased Ca^2+^ influx and pyroptosis. Consistent with this notion, treatment of mice with solutions containing high levels of Mg^2+^ was sufficient to protect mice from LPS-induced septic shock, the exact mechanism, however, still remains unexplored (58).

The above described STING (TMEM173)-dependent Ca^2+^-flux during bacterial endotoxemia occurred independently of the canonical cGAS pathway (*57.*) The canonical, dsDNA-activated cGAS-STING pathway, can induce NLRP3-Caspase-1 inflammasome activation as well, as a result of STING mediated lysosomal damage and K^+^ efflux (59). However this activation route is dependent on signal strength and cell type. It is functional in primary human monocytes, but not in macrophages and mouse embryonic fibroblasts, most likely as a way to limit large amount of pro-inflammatory cell death during antiviral responses (59).

### Cellular Death

The next step of pyroptosis is the cell committing to die. Pore formation is correlated with a loss of mitochondrial membrane potential (MMP), which is not due to cellular rupture as the loss of MMP still occurs in cells where lysis is prevented with the osmoprotectant glycine. This process is dependent on GSDMD pores as GSDMD knock-out cells do not lose mitochondrial viability (42, 60). The loss in viability is likely caused by ion influx or a general loss of membrane potential rather than proteins leaving or entering the cell, as Vasconcelos et al. (42) described in a time-resolved single-cell analysis of pyroptotic cells that loss of MMP and Ca^2+^-influx occurred much earlier than uptake of small molecules like PI (670 Da). In their study, loss of mitochondrial viability was quickly followed by cellular swelling, a loss of lysosome stability and finally loss of nuclear integrity seen as nuclear rounding and condensation (42). Hence, cells undergoing pyroptosis are already dead before rupturing.

### Membrane Rupture

Cellular swelling is one of the features observed during pyroptosis, and hypertonic solutions have been described to rescue GSDMD-dependent cell death (61). This supports a model, in which a cell is losing its integrity and is passively rupturing due to osmotic pressure, resulting in the release of proteins such as LDH or the alarmins HMGB1 and galectin-1 (50, 62). Intriguingly, a study by Kayagaki et al. found that in reality, cell lysis is a process regulated by dedicated proteins (63). Cells deficient in the cell adhesion protein Ninjurin 1 (NINJ1) did not rupture even after mitochondrial death, despite formation of GSDMD pores, release of IL-1β, and display of the typical balloon morphology. NINJ1 is not an exclusive regulator of pyroptosis as LDH release by apoptotic triggers is also impaired in NINJ1-deficient cells, implicating NINJ1 as a key cell lysis regulator in several cell death pathways. NINJ1 expression was recently shown to be stimulated by oxidative stress, making loss of mitochondrial viability and mitochondrial ROS production potential triggers of its activation (64). How exactly NINJ1 mediates cell rupture and whether NINJ1 is activated downstream of all gasdermins need further study. Interestingly, membrane rupture is not only important for the release of inflammatory mediators but also for the clearance of intracellular bacteria. Of note, pyroptotic cells trap viable bacteria within ruptured cellular debris, and these structures are termed pore-induced cellular traps (PITs) (65). Subsequently, PITs are removed by recruited phagocytes resulting in the clearance of the dead cell as well as the contained bacteria (66).

One open question remains, how some cells maintain IL-1β secretion without undergoing pyroptosis (67). For example, dendritic cells activated with certain oxidized lipids generated during tissue injury, can maintain IL-1β secretion for several days without cellular rupture (68). Neutrophils can also maintain IL-1β secretion without undergoing pyroptosis (69). It will be interesting to see in future studies whether this cellular integrity is maintained by increased ESCRT-mediated membrane repair, prevention of mitochondrial damage or other processes.

## Posttranslational Modifications Regulate Gasdermin Activity

### Phosphorylation of Gasdermins

Pyroptosis is a highly inflammatory and extremely rapid process and, hence, gasdermins need to be tightly regulated in their activity. One effective means of regulating fast cellular processes is by post-translational modifications (PTM), which have been shown to also regulate other, upstream steps of inflammasome activation (70). Phosphorylation is the best studied PTM. It could theoretically control the activity of gasdermins directly by modifying them or indirectly by modifying their interacting partners. An unbiased proteomic screen showed that phosphorylation of substrates can alter their cleavage by caspases-3, -7 and -8, which are all enzymes that can also cleave GSDMD and GSMDE (71–73). Evidence so far for phosphorylation exists only for Thr8 and Thr6 of human GSDMA and GSDME, respectively (**Figures 1B, D**). Both phosphorylation sites block gasdermin oligomerization and pore formation (74). Mechanistically, they likely block interaction between gasdermin monomers, *via* changing the charge of the first alpha-helix of the N-terminal domain that is critical for oligomerization (40). The serine-threonine kinase Polo-like kinase 1 (PLK1) mediates GSDMA phosphorylation, but whether it phosphorylates Thr6 in GSDME remains unexplored (74, 75). Whether the functions of other gasdermins are regulated *via* direct phosphorylation is unknown although all contain at least one serine or threonine residue in the first alpha-helix. Additionally, a phosphorylation site prediction tool has pointed to several potential phosphorylation sites for gasdermin D and E located in their linker regions. Future studies will ascertain whether the predicted sites are indeed phosphorylated and how these PTMs influence the nearby cleavage sites. The necroptosis-associated pore-forming protein MLKL shows a similar dependency on phosphorylation *via* RIPK3-dependent signaling before pore formation and subsequent necroptosis. Hence, phosphorylation of pore-forming proteins could be a common mechanism by which cells regulate pore formation *via* of death effector proteins (76).

Phosphorylation of gasdermin-interacting partners also may play an important, additional role as a means to regulate gasdermin activity. Phosphorylation of the apoptosis-associated caspases-3, -7 and -8 has been shown in several reports to regulate their activation and/or substrate recognition (77–80). Interestingly, the only known phosphorylation site on caspase-1 at Ser376 is also needed for its activation; whether caspase-4, -5 or -11 are phosphorylated is unknown (81). This raises the question whether the threshold of activation for caspase-4, -5 or -11 is set low, or the phosphorylation of these pyroptosis-associated caspases remains unexplored in sufficient detail. The N-terminal domain of gasdermins inserts into membranes *via* binding to lipids such as phosphatidylinositol phosphate on the inner membrane leaflet or cardiolipin present on the inner mitochondrial membrane or plasma membrane in mammalian and bacterial cells respectively (39, 40). Interestingly, GSDMD-NT as well as GSDMA-NT and GSDMA3-NT are only capable of binding to membrane lipids when phosphorylated, identifying membrane lipid composition and phospho-modifications as another control mechanism in the process of pyroptosis (45).

### Other Posttranslational Modifications of Gasdermins

Macrophages and dendritic cells stimulated with inflammatory stimuli such as LPS are known to switch their metabolic profile from oxidative phosphorylation to aerobic glycolysis (82). A study by Humphries et al. (83) recently discovered that one metabolic intermediate of this pathway, fumarate, can irreversibly bind to GSDMD at Cys191 (human)/Cys192 (mouse) and GSDME at Cys45 (mouse) in a process termed succination (**Figures 1C, D**). GSDMD Cys191 is located next to Leu192, which is the contact point for the C-terminal GSDMD domain responsible for autoinhibition (45). As mutation of Leu192 blocks binding of GSDMD-NT to membrane lipids, succination at Cys191 likely confers a similar effect on GSDMD. This prediction was confirmed by cysteine-modifying drugs, which blocked pyroptosis and death in an animal model of lethal endotoxemia (13, 83). Although speculative, a model emerges whereby the metabolic switch—i.e., oxidative phosphorylation to aerobic glycolysis—specifically blocks gasdermin pore formation by succination. Quite likely, this is only a part of a much broader effect caused by the reactive nature of fumarate, as multiple cysteine residues are modified in both GSDMD and GSDME (83). A model that metabolic switch influences gasdermin-mediated death was also supported by a recent study in which prolonged LPS stimulation of macrophages lead to an accumulation of the cell metabolite itaconate. Mass spectrometry analysis revealed, that itaconate directly bound to GSDMD at Cys77. This modification blocked caspase-1-dependent GSDMD cleavage and conferred tolerance to extended periods of LPS exposure (84). Another study observed that chemotherapy modified human GSDME by palmitoylation at Cys407 and Cys408 *via* the palmitoyltransferases ZDHHC2, 7, 11 and 15 (**Figure 1D**) (85). These modifications led to a decreased interaction of GSDME-NT with GSDME-CT after GSMDE cleavage by caspase-3, which facilitated the release of cleaved GSDME-NT and, thereby, pore formation (85). Inflammasome stimuli are known inducers of mitochondrial ROS, and ROS production is required for pyroptosis (86). One recently described mechanism suggested regulation of GSDMD by ROS *via* direct oxidation of human GSDMD at Cys38, Cys56, Cys268 and Cys467 (**Figure 1C**) (87). Indeed, mutation of these residues reduced GSDMD cleavage by caspase-1 and pore formation, supporting the idea that GSDMD oxidation is an important regulator of its function.

Only a few PTMs are currently described to influence gasdermin activity, although a high amount of phosphorylations and ubiquitylations are predicted. The necroptosis-associated protein MLKL, its upstream activators RIPK1 and RIPK3 as well as multiple inflammasome components are known to be ubiquitylated (88, 89). This, in combination with a wide range of predicted ubiquitylation sites, makes it likely that future studies will uncover gasdermin regulation *via* ubiquitin by influencing either its activation or degradation. Currently, the PTM landscape of gasdermins is sparsely described, with only few interaction partners and locations known. An increasing amount of research on PTMs will hopefully help to answer which upstream processes lead to these modifications, which enzymes catalyze the addition or removal of modifications, and how these modifications are hierarchically ordered to inhibit or activate gasdermin-mediated pore formation.

## Therapeutic Strategies to Block Gasdermin-Mediated Cell Death

**Figure 2:**
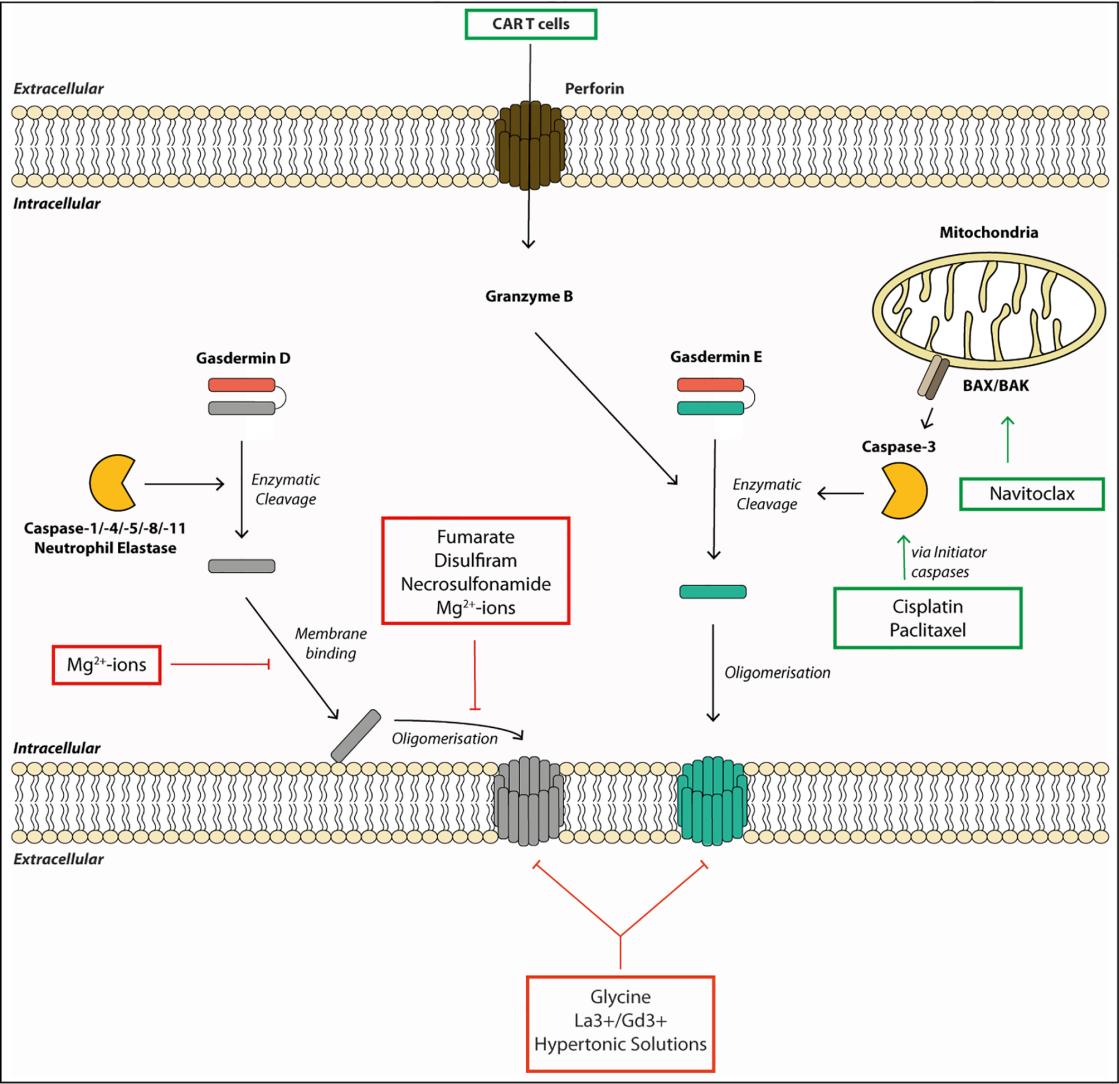
Gasdermin pore formation and cell lysis require multiple steps and can be targeted by therapeutics. Gasdermins are activated by enzymatic cleavage by proteases such as caspases or neutrophil elastase. This liberates the pore-forming N-terminal fragment (GSDM-NT). The GSDM-NT binds to phospholipids on the inner membrane leaflet. GSDM-NT then oligomerizes to form a membrane pore allowing the efflux of small proteins and ions across the membrane. Gasdermin pores eventually lead to cell death and membrane rupture. Some drugs promote gasdermin-mediated cell death. For example, chemotherapeutic drugs, such as Cisplatin, Paclitaxel or Navitoclax can initiate gasdermin E cleavage. They activate the initiator caspases, which, in turn, lead to gasdermin E cleavage by activating the executioner caspase-3. Other drugs or small molecules can block gasdermin-mediated cell death. For example, the membrane binding and the oligomerization step of gasdermin D can be blocked by Mg^2+^-ions by an unknown mechanism. Fumarate, Necrosulfonamide and Disulfiram can block oligomerization of gasdermin D by modifying Cys191. Finally, membrane rupture can be blocked by the osmoprotectant glycine, hypertonic solutions or the lanthanide ions La^3+^ and Gd^3+^.

Current therapies against aberrant inflammasome activation use IL-1-targeting drugs, such as the IL-1 receptor antagonist Anakinra (90), or direct inhibition of NLRP3 by specific inhibitors, such as MCC950 (91), and several others (92). As additional pro-inflammatory alarmins are released *via* the gasdermin pores and multiple inflammasomes can lead to gasdermin activation, gasdermin-specific drugs are highly desirable therapeutics. The extracellular addition of the amino acid glycine can inhibit gasdermin-mediated cell rupture, but it cannot prevent intracellular processes leading to cell death or IL-1β secretion (**Figure 2**) (38, 49, 60). The protective effect of glycine is dependent on its carboxyl group but is relatively unspecific, as glycine acts as an osmoprotectant and not as a specific inhibitor of gasdermin pore formation (93). Other described inhibitors such as lanthanides, Mg^2+^-ions and hypertonic solutions likely act *via* similar unspecific mechanisms, although Mg^2+^ ions block gasdermin oligomerization by blocking Ca^2+^-influx needed for pore formation (48, 58, 61). It is noteworthy, that the inhibitory concentration of magnesium needed to inhibit pyroptosis in macrophages exceeds the physiological extracellular concentration of magnesium by 10-fold, and that inhibitory effects were seen with nearly all divalent cations (58). Rathkey et al. (94) reported that the cysteine-modifying drug necrosulfonamide (NSA) blocked pyroptosis in human and mouse cells by binding to Cys191 (human)/Cys192 (mouse) of GSDMD and, thereby, blocked its oligomerization. NSA inhibition is not selective to gasdermins, as NSA blocks necroptosis by a similar mechanism: NSA binds to Cys86 of the pore-forming protein MLKL in humans to prevent oligomerization (95). Other groups found that cysteine-reactive drugs such as NSA and Bay 11-7082 rather inhibit pyroptosis by modifying upstream caspase-1 cleavage and not GSDMD itself, and that NSA can block IL-1β secretion even further upstream by inhibiting LPS-mediated priming (13, 96). Hu and colleagues (13) discovered in their compound screening that another cysteine-reactive drug, disulfiram (used in people to treat alcohol abuse), potently blocked GSDMD-mediated pyroptosis by the same mechanism as NSA, with no activity on other gasdermins or MLKL-mediated cell death (13). Disulfiram was described much earlier to oxidize thiols present in caspases and, thereby, blocked their substrate cleavage activity (97). And in a more recent study, Wang et al. (98) showed that disulfiram’s inhibitory effect is not restricted to GSDMD only, but can also inhibit GSDME. Although disulfiram is an attractive anti-pyroptotic drug, particularly because it is widely used in humans, the exact targets of disulfiram need to be better defined owing to its highly cysteine-reactive nature. Regardless, both NSA and disulfiram block GSDMD oligomerization *via* modifying Cys191, making either this site or the first alpha-helix (needed for pore formation) an attractive target for designing a more specific drug in the future.

While most drugs focus on blocking detrimental pyroptosis mediated by GSDMD, in some cases, boosting pyroptosis can be beneficial. For example, induced activation of GSDME has beneficial effect in treating various cancers because it promotes a more inflammatory tumor milieu (24). In a synthetic approach, Wang et al. (99) showed that tumor cells treated with engineered nanoparticle-GSDMA3 conjugates led to increased cell death and a significant reduction in tumor burden upon release of GSDMA3 from the nanoparticle. Chemotherapeutic drugs such as Cisplatin or Paclitaxel can induce activation of the initiator caspases-8 and -9, resulting in caspase-3 activation. Caspase-3 activation drives GSDME cleavage and pyroptosis, and makes this pathway an attractive target for tumor treatments (24, 85, 100). These are systemic drugs and should be used with caution, as systemic GSDME activation can have detrimental effects. For example, cytokine-release syndrome in patients treated with CAR T cells was GSDME-dependent. Granzymes released from these cells led to caspase-3 and -7 activation, and ultimately GSDME cleavage (23). As granzyme A and B directly cleave GSDMB and E, respectively, targeted delivery of these enzymes poses an interesting therapeutic option to treat immunosuppressive tumors, yet their administration will have to be tightly titrated or targeted to avoid detrimental side effects (21, 22). In conclusion, gasdermins are newly emerging targets for therapeutic targeting for positive and negative modulation of cell death and the resulting immune responses.

## Author Contributions

FF, KC, and JB drafted the manuscript. KC and JB supervised and edited the manuscript. All authors contributed to the article and approved the submitted version.

## Funding

JB is supported by Kennedy Trust, KTRR start-up fellowship (KENN 15 16 06), and MRC New Investigator Grant (MR/S000623/1). FF is supported by a Kennedy Trust, KTPS Studentship. KC is supported by National University of Singapore Start Up grant and a Ministry of Education Inauguration Grant.

## Conflict of Interest

The authors declare that the research was conducted in the absence of any commercial or financial relationships that could be construed as a potential conflict of interest.
